# Glycoprotein- and Lectin-Based Approaches for Detection of Pathogens

**DOI:** 10.3390/pathogens9090694

**Published:** 2020-08-24

**Authors:** Sammer-ul Hassan, Ahmed Donia, Usman Sial, Xunli Zhang, Habib Bokhari

**Affiliations:** 1 Mechanical Engineering, Faculty of Engineering and Physical Sciences, University of Southampton, Southampton SO17 1BJ, UK; xl.zhang@soton.ac.uk; 2 Biosciences Department, Faculty of Science, Comsats University Islamabad, Islamabad 45550, Pakistan; ahmeddonia123@yahoo.com (A.D.); usmansial@hotmail.com (U.S.)

**Keywords:** glycoproteins, lectins, pathogens, biosensors, miniaturization, diagnostics, nanotechnology

## Abstract

Infectious diseases alone are estimated to result in approximately 40% of the 50 million total annual deaths globally. The importance of basic research in the control of emerging and re-emerging diseases cannot be overemphasized. However, new nanotechnology-based methodologies exploiting unique surface-located glycoproteins or their patterns can be exploited to detect pathogens at the point of use or on-site with high specificity and sensitivity. These technologies will, therefore, affect our ability in the future to more accurately assess risk. The critical challenge is making these new methodologies cost-effective, as well as simple to use, for the diagnostics industry and public healthcare providers. Miniaturization of biochemical assays in lab-on-a-chip devices has emerged as a promising tool. Miniaturization has the potential to shape modern biotechnology and how point-of-care testing of infectious diseases will be performed by developing smart microdevices that require minute amounts of sample and reagents and are cost-effective, robust, and sensitive and specific. The current review provides a short overview of some of the futuristic approaches using simple molecular interactions between glycoproteins and glycoprotein-binding molecules for the efficient and rapid detection of various pathogens at the point of use, advancing the emerging field of glyconanodiagnostics.

## 1. Introduction

Microorganisms, such as bacteria and viruses, are widely found in the environment, marine and estuarine waters, soil, food, and the intestinal tracts of humans and animals. It is estimated that infectious diseases cause about 40% of the approximately 50 million total annual deaths worldwide. Waterborne pathogens cause 10–20 million of these deaths and further non-fatal infections of more than 200 million people each year [1,2]. Rapid and highly sensitive pathogen identification is crucial for human healthcare and subsequent therapeutic treatments, as well as for the prevention and control of epidemics and pandemics. The recent emergence of COVID-19 (caused by severe acute respiratory syndrome coronavirus 2 (SARS-CoV-2)) as a global public health concern and pandemic requires extensive testing, active monitoring and surveillance, and comprehensive data gathering to understand the intricate communal and global transmission patterns and to stop its spread [3,4].

Across much of the developing world, communicable diseases, such as malaria, respiratory infections, diarrheal diseases, and HIV/AIDS, constitute the most significant component of the total disease burden. In these places, the control of infectious diseases remains a critical challenge for health services. In Southeast Asia, the contagious diseases remain the major health problem; within the developing world, countries, such as China, Malaysia, Thailand, India, and Pakistan, are undergoing a transitional phase where they suffer a double burden from both communicable and non-communicable diseases. Infectious diseases pose a serious threat to the lives of millions of people across the world, especially vaccine-preventable diseases, such as diphtheria, invasive *Haemophilus influenza* disease, invasive meningococcal disease, invasive pneumococcal disease, measles, mumps, pertussis, poliomyelitis, rabies, rubella, tetanus, diarrheal diseases, and coronaviruses.

The majority of existing pathogen-detection assays employ direct pathogen detection, mostly achieved using PCR-based techniques [5], antibody-based methods, which include enzyme-linked immunosorbent assays (ELISA) [5] or fluorescent antibody assays [6]. These techniques lack the required sensitivity and specificity for low-concentration pathogen detection. Therefore, the more costly and laborious PCR assays are often necessary to enhance these screenings. Since these diagnostic methods are generally cumbersome and often have limited sensitivity, a variety of new pathogen-detection methods, including microcantilevers [7], evanescent wave biosensors [8], immunosorbent electron microscopy [9], and atomic force microscopy [10], have been investigated. However, these new techniques are unable to discriminate between closely related pathogen species and serovars/types with a reasonable sample throughput. Therefore, there is an unmet need for rapid, reproducible, and sensitive means of detecting these pathogens that place substantial burdens on human and animal health.

The inequities in health status and disease burden reflect the fact that primary healthcare infrastructure is lacking in the world’s least developed countries. Therefore, research aimed at rapid, simple, economical, and straightforward diagnostics for infectious diseases is needed to manage the problem. The science of glycoproteins holds enormous potential that can be exploited by using the millions of unique tags (“business cards”) of pathogens and their serovars/types. Many health-related issues can be addressed through timely diagnosis with the help of molecular biology and miniaturization techniques at the point of use. Therefore, it is imperative for us to re-examine our current health-related research and practices to improve our existing diagnostic and healthcare delivery systems.

**Figure 1:**
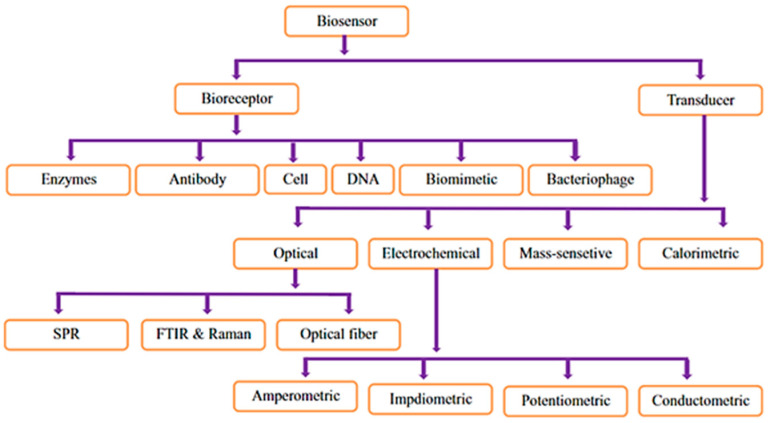
Example of bioreceptors and transducers [15].

Among the various principles and techniques used for the detection of microbes, biosensors are emerging as promising tools for this purpose. Biosensors are analytical devices that convert biological responses into electrical, optical, or mass-sensitive signals. Biosensors use output elements to obtain the quantity of the target molecules [11]. Over time, biosensors have increased in importance due to advantages, such as rapid real-time detection, portability, ease of use, and multi-pathogen detection in field and laboratory analyses [12]. A typical biosensor usually consists of a bioreceptor, transducer, and an output unit. Examples of bioreceptors and transducers are shown in Figure 1. Specific biosensors may be constructed by exploiting a multitude of biorecognition elements and transducers that have particular advantages and limitations. The most well-developed interactions used for the construction of biosensors include enzyme–substrate, antibody–antigen, DNA–DNA, and aptamer–target. The transducer unit may consist of electrochemical or optical sensors or a combination of both. Electrochemical biosensors are preferred over other transducing systems as they can detect even 10^−7^ to 10^−9^ M, or 30 ppb, gaseous compounds [13]. On the other hand, peptide biosensors use peptides for molecular detection, making use of the specific binding sites on the target molecule. Such biosensors have shown potential, and the exploitation of protein biomarkers is extensively used for the monitoring and diagnosis of various diseases like cancer, tuberculosis, human immunodeficiency virus, microbial infections, and pregnancy screening [14].

Given the current and future challenges in diagnostics and medical sciences, advances in glycoprotein-based biosensors have the potential to lead to cost-effective, timely, and accurate diagnostic tools. Development of such devices would be a milestone, highlighting the need more effort and research to understand and develop practical approaches to exploit these biomolecules.

## 2. Glycoproteins

The term “glycomics” (the study of carbohydrates and their role in systems biology) has been frequently used in the literature in recent years, suggesting this to be an important field in our developing understanding of systems biology. Little health research is currently underway using glycoproteins as diagnostic markers for the rapid identification of infectious agents, as can be gauged from the literature citing relevant work published in the last ten years [16].

**Figure 2:**
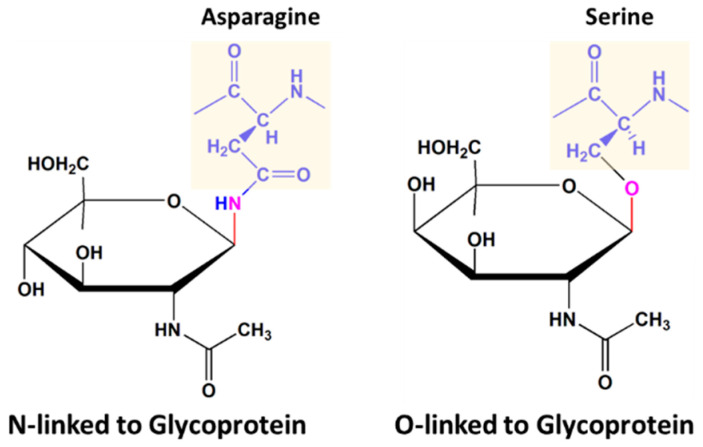
Schematic of the difference between O- and N-linked glycosylation.

Glycoproteins are formed as a result of post-translational modification, a group of chemical modifications that alter the physicochemical properties of membrane-bound or secreted proteins, e.g., folding, stability, activity, immunogenicity, proteolytic resistance, local structure, and protein solubility. Therefore, these modifications play essential roles in the regulation of various intracellular processes. Glycosylation is one of the most complex types of a post-translational modification. It is a process in which carbohydrate units attach covalently to the backbone of a protein at asparagine, hydroxylysine, serine, or threonine residues, thus making it a glycoprotein [17]. Glycosylation is involved in about 50–80% of cellular processes, and glycoproteins represent approximately 70% of the therapeutic proteins approved by European and U.S. regulatory agencies for clinical or pre-clinical development [17]. Most of the time, glycosylation occurs via either the N-linked glycosylation pathway or the O-linked pathway. In the N-linked process, glycans are conjugated to the side-chain amide nitrogen of asparagine in Asn–X–Ser/Thr consensus sequences, where X can be any amino acid other than proline. O-linked glycosylation involves the hydroxyl group of a threonine or serine residue [18]. Figure 2 shows the difference between O- and N-linked glycosylation.

This review discusses the importance of glyco-processes, particularly O- and N-linked glycosylation, in the rapid detection of a range of infectious disease agents, and applications of glyconanotechnology to enhance these efforts. Initially, N-linked glycosylation was thought to occur only in eukaryotes. Later, N-linked glycosylation was found to occur in archaea and bacteria, which show more diversity than eukaryotes in terms of carbohydrate units attached to proteins [19]. In prokaryotes, these glycosylated proteins may help with host-cell invasion and pathogenicity [20]. Any aberrance in the glycosylation mechanism can compromise the healthy survival of an organism. Many glycoproteins exist among emerging and re-emerging pathogens, including viral and bacterial pathogens with well-defined N- or O-linked sugars through which they interact with host cells. The current review provides detailed insight, including some examples where knowledge obtained from experimental work can potentially be used to develop cost-effective and robust glyconanodiagnostic technology platforms for infectious diseases.

The immobilization of carbohydrates has facilitated the development of effective studies of carbohydrate interactions, allowing scientists to examine the effects of specific sequences in a format intended to mimic natural glycan presentation, leading to a deeper understanding of different cellular processes. Efficient immobilization of sugars onto appropriate solid surfaces is a prerequisite for successful preparation of carbohydrate microarrays. The idea is that immobilized mono- and oligosaccharides act similarly to cell-surface carbohydrates, and so they are recognized and subsequently bound by biomolecules of interest. A deep and detailed understanding of carbohydrate–protein interactions will eventually lead to the development of improved diagnostic tools and better therapeutics.

## 3. Molecular Signatures and Identification Tags

Glycoproteins display various interactions between proteins and glycans that play roles in organizing molecular structures and become molecular signatures for specific protein and glycan moieties. These glycoproteins have oligosaccharide chains that act as identification tags that can be used to label proteins at the outer surface of the cell. These oligosaccharide chains are attached to the protein through either N- or O-glycosidic bonds. Lectins, mucins, and several polypeptide hormones are glycoproteins. Glycolipids are carbohydrate-containing lipids with a sphingosine backbone. Certain animal diseases are characterized by abnormal quantities of glycolipids in the central nervous system. Carbohydrate residues of membrane-bound glycolipids and glycoproteins are located on the exterior surfaces of cells. The glycoproteins play vital roles in various biological processes, like cell-to-cell recognition, growth, differentiation, and apoptosis. Specific N- and O-linked glycoprotein changes are associated with the development and progression of various diseases, and therefore have the potential to be used as tags for disease diagnosis and prognosis [21]. On the basis of reported data, a particular O-linked glycoprotein, β-N-acetylglucosamine (O-GlcNAc), has been established to be involved in the etiology of diabetes [22], breast cancer [23], and many other diseases. As bacteria can produce both O- and N-linked glycoproteins [24,25,26,27,28,29], there is considerable interest in harnessing this potential to produce glycoconjugates and develop rapid and economical glyconanodiagnostic methods.

The process of translating glycoprotein discovery and the development of diagnostics and therapeutics into the clinical environment will require an in-depth understanding of the biosynthetic pathways involved, as well as of the roles that these molecules play, in host–cell interactions. As most of these glycoproteins are exposed on the cell surface, the O-linked glycoproteins occur in various forms with a high degree of variation between and within species, which makes them more suitable and promising for use as markers for rapid identification. O-glycosylation is more common in bacteria, whereas N-glycosylation is more common in archaea. Gram-negative bacteria widely exhibit oligosaccharyltransferase-mediated (OST) O-glycosylation, whereas Gram-positive bacteria have not experimentally shown this type of glycosylation. Gram-positive bacteria significantly exhibit cytoplasmic O-glycosylation, which can be attributed to the alternative OST O-glycosylation pathways [30].

### 3.1. Bacterial Pathogens

Given the persistence and continuing threat of bacterial pathogens, further insight into bacterial glycoproteomics is required to advance diagnostic and therapeutic medicines to combat infectious diseases. Reports have increased of both O- and N-linked protein glycosylation pathways in bacteria, particularly amongst the mucosal-associated pathogens, due to the advancement of analytical methods and genome-sequencing projects [19,20]. Just over 15 years ago, evidence of a pertinent N-linked protein glycosylation pathway was reported in the Gram-negative pathogenic bacterium *Campylobacter jejuni* [20]. Diagnostic approaches will exploit the unique carbohydrate structures that decorate the surface proteins of many pathogenic bacteria. The unique surface carbohydrates that decorate many surface proteins of bacterial pathogens have the potential to improve the accuracy of bacterial-infection diagnosis, differentiating not only between pathogen and host but also between individual species and strains of bacteria [31].

**Figure 3:**
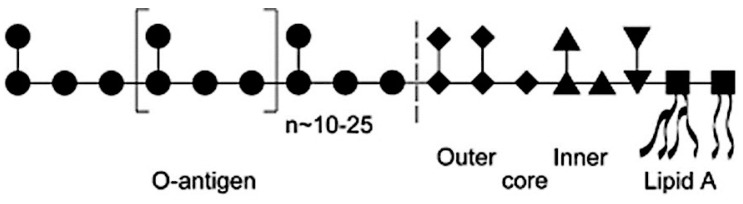
Schematic structure of an enterobacterial lipopolysaccharide molecule with different and diverse sugar residues. The lipids are depicted by curved lines, and the sugar residues are as follows: GlcN (▪), Kdo (▾), heptose (▴), hexose (◆), and O-antigen components (•), most commonly hexose. (figure is taken from Reference [33].).

Pathogens naturally synthesize glycoproteins and glycolipids with unique saccharides due to their wide diversity and complexity, through different combinations of linkages, stereo-isomerism, and repeated branching comprising various monosaccharide units. For instance, bacterial pathogens synthesize packages of glycolipids and glycoproteins that have particular functions and specific cellular locations. These may range from the serotype-specific configuration of uniquely distinct surface structures within a species, e.g., capsular polysaccharide (CPS) and lipopolysaccharides (LPS), as well as relatively conserved structures with the polymer of amino sugars attached to a glycophospholipid generally commonly present in Enterobacteriaceae, e.g., enterobacterial common antigen (ECA) [32]. The glycoproteins and glycolipids from bacterial pathogens displayed on the surface are involved in host–pathogen interaction, such as LPS, a major envelope component of Gram-negative bacteria, i.e., *Escherichia coli*. The O-polysaccharide antigen (O-antigen), comprising diverse sugar units exposed on the surface, is attached to a core oligosaccharide that is linked to the hydrophobic membrane anchor called Lipid A (endotoxin), collectively constituting LPS, as shown in Figure 3 [33].

The outer membrane of Gram-negative bacteria consists of O-antigen, which is a polysaccharide composed of multiple oligosaccharide units (two to six units). This type of polysaccharide exists in various orders and linkage schemes of sugars, and are therefore different within the same or across multiple species. The genes involved in O-antigen biosynthesis are generally found on the chromosome as an O-antigen gene cluster, and the structural variation of O-antigens is mirrored by the genetic variation seen in these clusters. O-antigen, due to its significant variability, is a useful identification tag for identifying different strains within species. For instance, *Vibrio cholerae* synthesizes more than 190 O-antigens, *Pseudomonas aeruginosa* synthesizes 20 O-antigens, *Salmonella enterica* synthesizes 54 O-antigens, and 181 O-antigens are synthesized by *E. coli* serotypes [33,34].

### 3.2. Viral Pathogens

Genetic and structural material of viral pathogens is generated via biosynthetic pathways, such as a surface protein, on a viral pathogen being modified by glycosylation pathways. N-glycosylation plays an important role in the glycosylation pathways used by viruses. Firstly, host cellular chaperones and folding factors are used, where folding and trafficking of surface protein is promoted by N-glycosylation. In most cases, calnexin or calreticulin are used. Glycosylation of viral protein is mostly higher than cell-protein glycosylation, which serves as a clear differentiator between host and viral proteins. Viral proteins become more complex during evolution, which affects folding, survival, and trafficking of the virus [35,36,37]. Secondly, variations in glycosylation make the virus more identifiable by immune cells than antibodies, which can allow further virus transmittance. Pathogenesis and immune evasion are potent functions linked to glycosylation in viruses, such as HIV, influenza, hepatitis, West Nile virus, etc.

The major reason for the emergence of new viruses is their ability to evolve by incorporating or losing glycosylation sites between closely related viruses, resulting in an alteration in protein folding and conformation, thereafter affecting receptor recognition and immune status. Many viral pathogens, such as HIV, hepatitis C virus (HCV), Dengue virus, etc., are surrounded by an outer envelope that mediates the interactions of these viruses with the receptors present as cellular entry points at their corresponding target cells during the infection process. The envelopes of both HIV and HCV contain a large number of surface-displayed glycans that help these viruses to escape the immune response of the host.

The emergence of drug-resistant strains of HIV and HCV, the associated side effects of the existing drugs, and drug unaffordability to low-income populations encourage continuous efforts to discover new anti-viral agents. The initial steps during the entry of HIV and HCV into the host cell involve the interactions of their outer envelopes with the cellular receptors [38]. The envelopes of both HIV and HCV are heavily glycosylated; roughly 50% of the mass of the surface envelope glycoproteins gp120 of HIV and E2 of HCV is accounted for by glycans. These glycans play essential roles in protein folding and immune evasion by the virus [39]. However, these are important therapeutic targets for carbohydrate-binding agents. Vigerust et al. [40] elucidated viral protein glycan structure, function, and host glycoprotein interactions. In this context, an approach that is exceptionally promising for identifying anti-viral agents involves targeting the glycan shield of the surface envelope proteins of these viruses to inhibit their entry to the host cell [41].

Severe acute respiratory syndrome coronavirus 2 (SARS-CoV-2) pneumonia is a newly discovered disease that spread progressively throughout Wuhan (Hubei province) to other regions in China and all over the world. The emergence of SARS-CoV-2 disease (COVID-19) in China at the end of 2019 led to a global pandemic [42]. Coronaviruses are enveloped viruses, with the viral structure primarily formed of structural proteins, such as spike (S), membrane (M), envelope (E), and nucleocapsid (N) proteins, and hemagglutinin-esterase (HE) protein in some betacoronaviruses, such as SARS-CoV-1 and SARS-CoV-2. The S protein is a heavily glycosylated protein that produces homotrimeric spikes on the surface of the viral particle and initiates entry of the virus into host cells. Hemagglutinin-esterase is a hemagglutinin similar to influenza virus hemagglutinin because it has the ability to bind to sialic acid on host cell-surface glycoproteins and has acetyl-esterase activity. Hemagglutinin-esterase may facilitate the entry and pathogenesis of coronaviruses that contain this protein in their viral structure [43]. Coronavirus infections are initiated by the spike glycoprotein, which attaches to host receptors and fuses the viral and cellular membranes. The S glycoprotein of coronaviruses mediates 9-O-acetyl-sialic acid (9-*O*-Ac-Sia) binding. In contrast, the HE protein acts as a receptor-destroying enzyme via sialate-*O*-acetyl-esterase activity to facilitate the release of viral progeny from infected cells and avoid binding to non-permissive host cells. The receptor-binding position is conserved in all coronavirus S glycoproteins which engage 9-O-acetyl-sialogycans, with a structure comparable to those of the ligand-binding pockets of coronavirus hemagglutinin-esterases and influenza virus C/D hemagglutinin-esterase fusion glycoproteins [44]. A study reported a model of the homo-trimer structure of COVID-19 spike glycoprotein in both open (ligand-bound) and closed (ligand-free) conformations, which play a role in host-cell adhesion. Researchers also predicted the distinct N- and O-linked glycosylation sites of the spike glycoprotein that differentiate it from coronavirus and underscores the protection and camouflage of coronavirus from the host defence system. Spike protein, which is the common target for neutralizing antibodies and vaccines, consists of two subunits: S1 and S2. The S1 subunit has a receptor-binding domain (RBD) that is involved in recognizing and binding with the cell-surface receptor. The S2 subunit is responsible for the fusion of cellular and virus membranes. The S1 domain of the coronavirus spike glycoprotein has the ability to strongly interact with human CD26, a key immunoregulatory factor for virulence and hijacking [45,46].

### 3.3. Lectins

Lectins, a class of sugar-binding and cell-agglutinating proteins are usually obtained from bacteria, plants, animals, virus, and fungi. Several carbohydrate-binding proteins known as lectins have been discovered that block viral entry to a host cell by specifically binding to the glycan fragments on the viral surface, thereby inhibiting viral infection. The most abundant sources of such lectins are different strains of algae [47]. For example, two of the most potent anti-HIV and anti-HCV lectins, cyanovirin-N and griffithsin, were isolated from blue-green algae *Nostoc ellipsosporum* and red algae *Griffithsia* sp., respectively [48,49]. These two lectins are at the pre-clinical stage for use as topical microbicides for the prevention of sexual transmission of HIV [50,51]. Another lectin, microvirin (MVN), inhibits HIV cellular infections with higher specificity and has been isolated from blue-green algae and *Microcystis aeruginosa*. Algae, therefore, represent a potential source of new anti-viral lectins.

**Figure 4:**
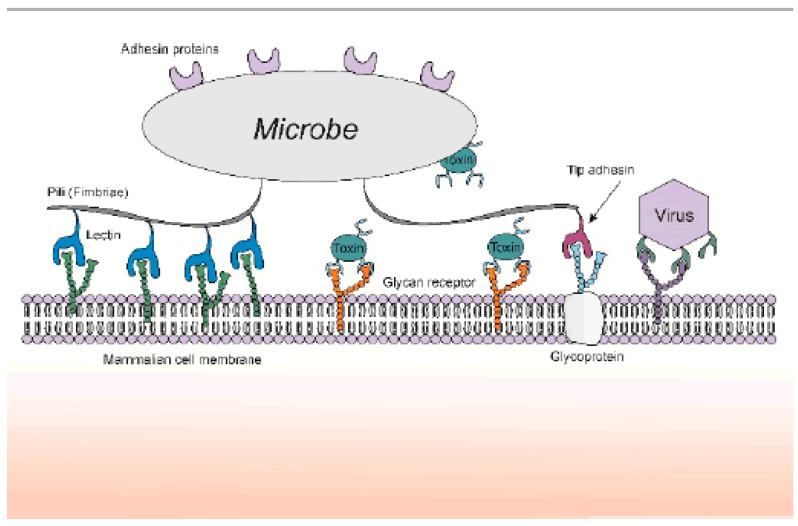
Nature of pathogenic agents binding to carbohydrate ligands on host cell surfaces (figure was taken from Reference [52]).

The ability of a pathogen to attach to host cells allows it to overcome the natural mechanical shear forces and escape immunological surveillance mechanisms. Bacteria, viruses, and toxins overcome low-affinity binding by combining interaction sites into multiple sequences. Interactions occur through adhesin binding to many copies of glycan receptors present on host cells. Adhesins are located either on microbial surfaces or external organelles, present in several hundreds of copies (Figure 4). Although the affinity of this interaction is relatively low, the unique ability of adhesins to bind to multiple glycan receptors on host cells allows bacteria to bind more effectively to host cells.

**Figure 5:**
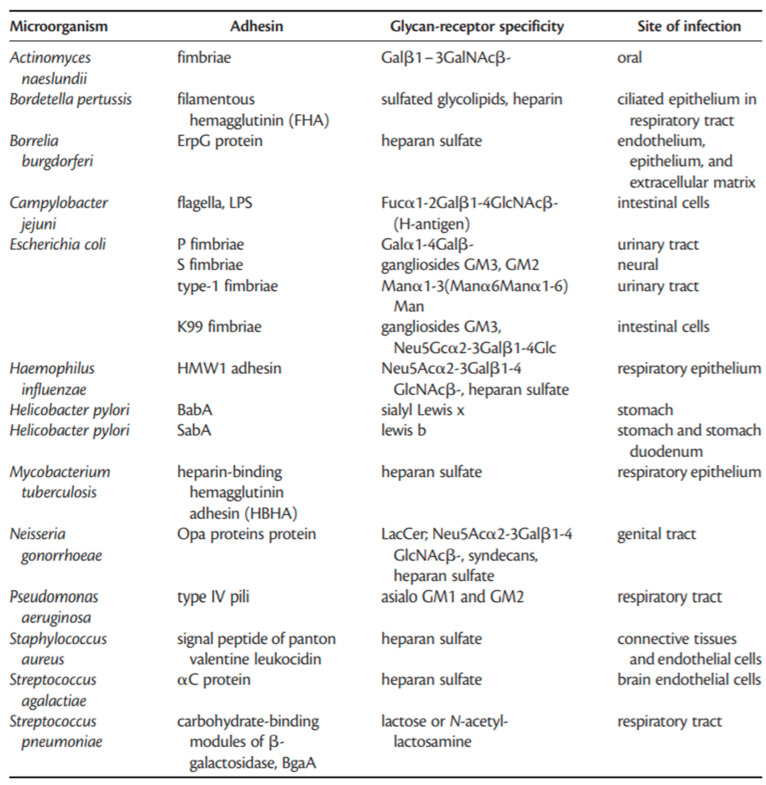
Examples of interactions of bacterial adhesins with glycans (figure was taken from Reference [56]).

Lectins are also present in cells and biological fluids. Several features of cell membranes have been revealed through studying these lectins [53,54]. Lectins have been reported to be involved in a variety of biological processes, such as cell–cell and host–pathogen interactions, serum glycoprotein turnover, and innate immune responses [55]. Bacterial lectins occur in the form of elongated, submicroscopic, multi-subunit protein appendages, which are known as fimbriae (hairs) or pili (threads) and which interact with glycoprotein and glycolipid receptors on host cells. Bacteria express 100–400 such appendages, having a diameter of 5–7 nm, which can extend to hundreds of nanometers in length. The carbohydrate-recognition domain of the lectin is found in the minor subunit, which is usually located at the tip of the fimbria. Some of the other bacterial lectins are monomeric or oligomeric membrane proteins [56]. Most bacteria (and possibly other microorganisms) have multiple adhesins with different carbohydrate specificities [55,57], and these carbohydrate signatures can be exploited for the specific identification of bacterial pathogens. Figure 5 provides examples of interactions of bacterial adhesins with glycans [56]. Each adhesion presented for a specific bacterium has the ability to bind to a specific carbohydrate moiety. For example, the P fimbriae and S fimbriae of *E. coli* can bind specifically to Galα1–4Galβ and gangliosides GM3, GM2 glycan moieties, respectively. Type IV pili situated on *Pseudomonas aeruginosa* are able to bind specifically to Asialo GM1 and GM2 in host cells. As shown in Figure 5, a large number of bacterial adhesins bind to specific carbohydrate moieties.

The primary function of bacterial lectins is to facilitate attachment or adherence to host cells; thus, they are also called adhesins, and glycan ligands on the surface of host cells are called receptors [54]. Cell-surface lectins serve for the attachment of the organisms to host cell-surface glycoconjugates in the initiation of infection [53,54,55]. Lectin–glycan interactions occur due to the interaction of their aglycones with hydrophobic side-chains close to the mannose-binding site of the subunit. Lectins are low-affinity binding molecules that can bind to several pathogens. A mono- or oligosaccharide would be enough to bind the specific pathogen. Their specificity, availability, vast range of molecular weights and stability make them beneficial for laboratory purposes [54].

Plant lectins that have shown anticoronaviral activity include mannose-, glucose-, galactose-, *N*-acetyl-glucosamine-, and *N*-acetyl-galactosamine-specific agglutinins. The mannose-specific plant lectins are the most prominent lectins with anti-coronavirus activity. There are 12 *N*-glycosylation sites in the SARS-CoV-1 spike protein. The sugars attached to four of these 12 *N*-glycosylation sites have been characterized [58]. Of these four glycosylation sites, two sites were found to be high-mannose-type glycans, whereas the other two exhibited complex glycan structures. The potent anti-SARS-CoV-1 activity of mannose-specific plant lectins can be attributed to the presence of high-mannose-type glycans. Two of the possible anti-viral interference targets of plant lectins in the replication cycle of SARS-CoV-1 have been identified. The first target identified is early in the replication cycle, most likely virus attachment, whereas the second is at the end of the infectious virus cycle [58]. Griffithsin, which is another high-mannose-binding lectin, impedes SARS-CoV-1 infection in vitro and in vivo via particular binding to the spike glycoprotein of SARS-CoV-1 and exhibits activity against multiple other coronaviruses pathogenic to humans, other mammals, and birds [59].

**Figure 6:**
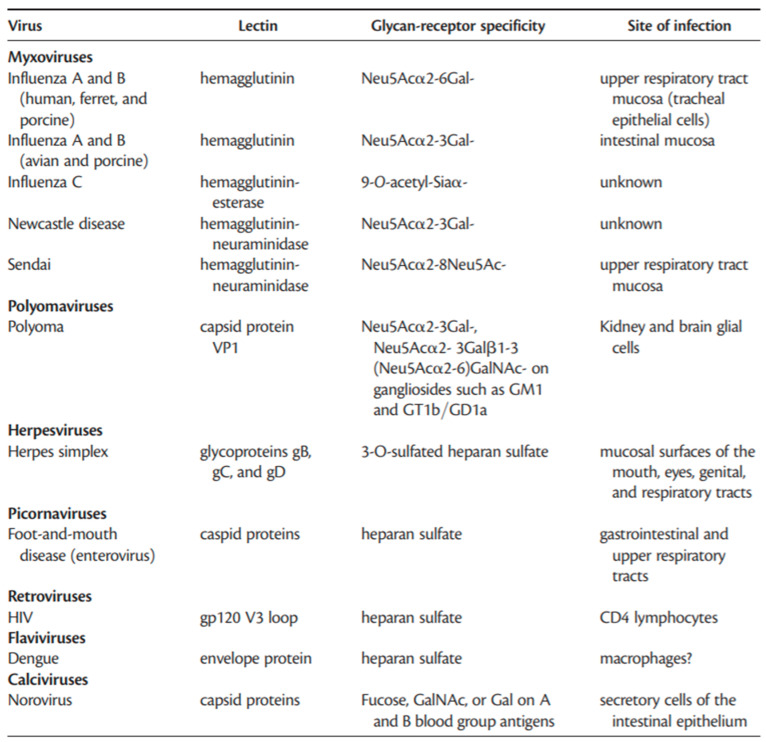
Examples of viral lectins and hemagglutinins (figure was taken from Reference [56]).

In addition, enveloped viruses, such as Sinbis and Venezuelan equine encephalitis (VEE), which have glycoprotein moieties on the surface, should also be amenable to capture via lectin probes. These glycoproteins contain N-linked saccharides of the high-mannose type [60] and therefore would be expected to bind to Con-A-based probes. Lectins have also been identified in viruses. Prior to the initiation of infection, the influenza virus requires attachment through haemagglutinin (a viral lectin) to sugars, usually sialic acid, present on host cells. Therefore, recognition of cell-surface carbohydrates is a prerequisite for invasion by lectin-carrying viruses [30]. Figure 6 lists examples of viral lectins and hemagglutinins. Each viral lectin presented for a particular virus has the unique ability to bind to a specific carbohydrate moiety. The influenza virus hemagglutinin binds to sialic-acid-containing glycans. Hemagglutinin of influenza A and B (human) can bind to the Neu5Acα2–6Gal glycan receptor in the upper respiratory tract mucosa, whereas the hemagglutinin of influenza A and B (avian and porcine) has the unique ability to bind to the Neu5Acα2–3Gal glycan receptor on the intestinal mucosa. Therefore, each viral lectin has the ability to bind to specific carbohydrate moieties.

### 3.4. Antibodies vs Lectins

Immunoaffinity-based techniques are often used for the identification and characterization of microorganisms, but an antibody is generally only useful for the isolation of a given strain or species, limiting the utility of this approach when the capture of several microbial species is desired [61]. In contrast, we tested the hypothesis that protein–carbohydrate binding pairs can provide broad-spectrum interactions to clean and concentrate microorganism samples. Lectins, carbohydrate-binding proteins of nonimmune origin, have been shown to capture microbial samples via glycoconjugates exposed at or near the cell surface. Such interactions were previously used to identify bacteria based on binding to a panel of lectins of varying carbohydrate specificity [62,63,64]. Many bacteria also have lectins present on their cell surfaces. These proteins termed microbial lectins or adhesins, mediate the interactions between the host-cell glycoproteins and pathogens at an early stage of infection [61]. Two examples of these proteins are the mannose-binding adhesion of fimbriated *E. coli* [65] and Lewis-b (Leb) binding [66].

Antibodies are often used in biosensor development for microbe identification due to their high specificity and strong interactions. The disadvantage of this assay format is the demand for antibodies against the antigens, which take more than three months to obtain (including immunization, purification, and shipment). Even if the purified antibodies against the desired antigens are obtained, cross-reactivity may occur, resulting in lower specificity. For the regeneration of a biosensor chip, powerful agents are needed due to the strong antibody–antigen binding.

**Figure 7:**
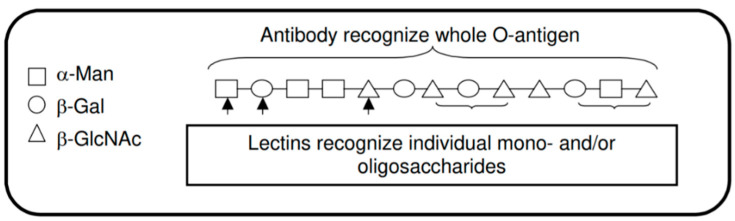
Antibody and lectin recognition of O-antigen (the figure was taken from Reference [67]).

Using lectins instead could solve these problems since several hundred lectins with different saccharide specificities are commercially available. Lectins are more stable but are less specific compared with monoclonal antibodies, which can be compensated for by applying an array of lectins in parallel. A high-affinity antibody only binds to the antigen for which it was designed and so can only be used to detect that antigen. In contrast, a low-affinity molecule (lectin) may bind several different ligands (pathogens). Figure 7 shows the difference between lectin and antibody recognition of O-antigen structures [67].

**Figure 8:**
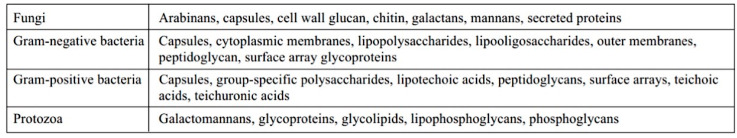
Lectin receptors of microorganisms (the figure was taken from Reference [67]).

Direct aggregation of suspended microorganisms is one of the important applications of lectins in microbiology. Almost all microorganisms express surface-exposed carbohydrates, which are potential lectin reactive sites. The lectin receptors of some microorganisms are listed in Figure 8 [68]. Lectins have a wide range of receptors that differ according to the organism. For example, lectin receptors in Gram-positive bacteria could include peptidoglycans, teichoic acid, and lipoteichoic acid. In contrast, in Gram-negative bacteria, receptors of lectins may include lipopolysaccharides, cytoplasmic membranes, and surface-array glycoproteins. Mannans, chitin, and arabinans are some of the lectin receptors in fungi. In protozoa, common lectin receptors include galactomannans, glycoproteins, glycolipids, lipophosphoglycans, and phosphoglycans.

The receptors commonly used in biosensors for pathogen detection are based on antibodies and nucleic acids. In the past decade, carbohydrates have been increasingly considered as alternative bioreceptors because they are present on almost all cells as polysaccharides, glycolipids, and glycoproteins, as well as other glycoconjugates, and they are involved in many biological processes [57]. In contrast to nucleic acids and antibodies, carbohydrates are more resistant against denaturation. Due to their small size, higher densities of carbohydrates per unit surface area are possible. This high carbohydrate density enables multivalent interactions, which enhance the binding affinity [69]. Mimicking the situations encountered on the cell surfaces during the initial recognition processes offers promise for the development of biosensors for the detection of carbohydrate-binding proteins and pathogens.

## 4. Biosensors

Biosensor techniques play an important role since they are based on biospecific interactions between the biological parts of the analyte (microorganisms) and the biosensor. Biosensors are based on the combination of biological receptor compounds (nucleic acids, lectins, enzymes, antibodies, etc.) and a physicochemical transducer directing the physical, and, in most cases, real-time observation of a particular biological event (e.g., glycoprotein, lectin–carbohydrate, and antibody–antigen interactions). Optical biosensors are promising, as they allow real-time and direct label-free detection of bacteria. Surface plasmon resonance (SPR) is a robust optical biosensing method that is used for non-label bioaffinity interaction analysis (e.g., antigen–antibody, lectin–saccharide, and receptor–hormone interactions).

The recognition and identification of glycans by other molecules with exquisite specificity and high affinity are the key current and ongoing developments in glycan-related basic research and clinically relevant therapeutic and diagnostic applications. Therefore, lectins as carbohydrate-binding proteins should be coated onto sensors to detect glycans. Bacteria, viruses, protozoa, and fungi express an enormous array of glycan-binding proteins (lectins). As a possible method to detect these lectins, glycans should be coated onto sensors to detect microbial lectins.

### 4.1. Glycan Biosensors

Glycan biosensors are based on the binding of lectin carbohydrates with glycoproteins. The process involves the attachment of carbohydrates to the solid surface and binding of glycoproteins in the whole-cell assays. Several strategies are used for presenting carbohydrates on the surface, such as conjugation methods, including binding of biotin to streptavidin or formation of self-assembled monolayers (SAMs), and noncovalent and covalent bond formation on the surface [70,71,72].

**Figure 9:**
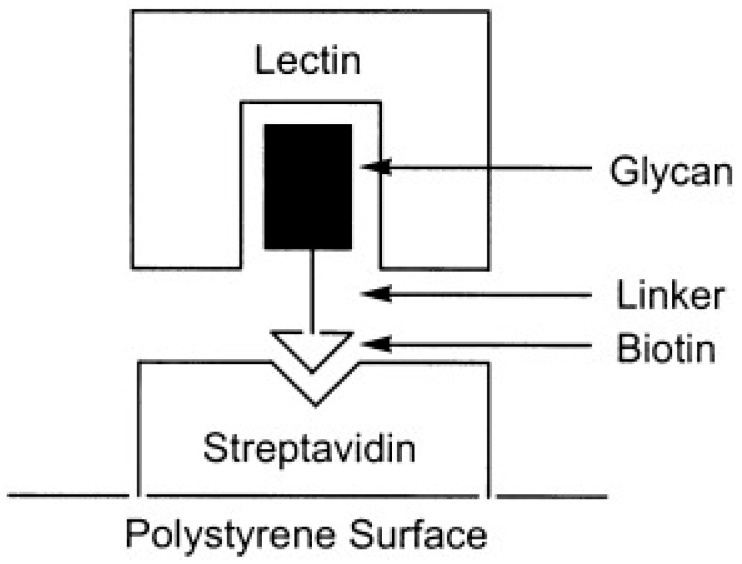
Surface display of carbohydrates for lectin-binding assays by the interaction of biotin with streptavidin (figure taken from Reference [70]).

The first method depends on the natural binding affinity of biotin for streptavidin [71]. Figure 9 explains the surface display of carbohydrates for lectin-binding assays via making use of the interaction of biotin with streptavidin [70]. The previous study reported that streptavidin-biotinylglycans could be used as a tool for characterization of oligosaccharide-binding specificity of lectins [73]. They biotinylated the anomeric asparagine residues of twelve glycans and immobilized them onto a 96-well titer plate coated with streptavidin for characterizing of the binding properties of six common lectins including concanavalin A, wheat germ agglutinin, *Phaseolus vulgaris* (red kidney bean) erythroagglutinin, *Lens culinaris* (lentil), *Datura stramonium* agglutinin, and *Sambucus nigra* (elderberry bark) agglutinin.

The second method is the formation of SAM of alkanethiols on a gold-coated substrate, which can help in displaying of densely packed structures on a surface [74]. The characteristic features, including reactivity of a monolayer, are relied on the exposed functional groups. The previous study reported that hydroxylated surfaces, which have the possibility of derivatizing the OH groups with biologically active moieties, present a readily derivatized moiety for the attachment of biomolecules like a thiol-terminated hexasaccharide [75]. N-acetyl glucosamine groups presented on the surface can be glycosylated when incubated with galactose and GalTase. This glycosylation pattern can be recognized through lectin binding to the newly formed disaccharide [70]. It is widely known that SAMs can have a role in the generation of a carbohydrate array. This can be achieved via immobilization of a series of cyclopentadiene-functionalized monosaccharides on a SAM displaying reactive quinone groups. Formation of rapid and irreversible Diels—Alder reaction between the cyclopentadiene and quinone groups leads to localization of each monosaccharide, allowing for interrogation with carbohydrate-binding proteins [70].

Both biotin-streptavidin- and SAM-based methods for biomolecule conjugation have been successfully used for the presentation of carbohydrates on a surface. In each case, incubation of the surface with various glycoproteins has confirmed known carbohydrate-binding specificities [71,72]. The biotin-streptavidin method uses the principle of natural binding affinity of biotin to streptavidin whereas SAM is based on the formation of alkane thiolate monolayers on a gold-coated substrate which permits the display of densely packed structures on a surface. For the biosensor development, biotin–streptavidin-based carbohydrate presentation is limited to the display of a unique substrate on each individual surface and requires physical separation of different biomolecules, typically using a 96 well enzyme-linked immunosorbent assay (ELISA) plate. In contrast, SAMs show potential in the synthesis of carbohydrate arrays and are particularly suited to the construction of biochips due to the ease of their construction, increased inertness to protein adsorption and demonstrated utility in conjunction with cell cultures [76,77]. Consequently, the simultaneous detection of carbohydrates by the binding of lectin glycoprotein in the context of a monosaccharide-based array on biochips will be an important development in the emerging field of glycomics [40].

The noncovalent bond formation may be exploited for microarray formation. Still, most of the reported techniques for immobilizing mono- and oligosaccharides to solid surfaces are based on covalent bond formation between the sugars and the carrier material. The process of microarray formation is complicated due to the vast range of potential substrates, variety of surface-coating methods, and the selection of a proper linker molecule followed by a chain of chemical reactions that leads to the formation of a stable microarray. Noncovalent immobilization of sugars for carbohydrate microarray production is a simple and faster process. This approach is beneficial when the noncovalent binding is strong enough to yield stable surfaces capable of withstanding the necessary washing steps, and the incubation conditions in biomolecule–sugar interaction studies on microarrays. Its relative simplicity gives this approach certain advantages over the more complex covalent immobilization methods. The two main subsets of this particular immobilization technique use chemically unconjugated and conjugated sugars [78]. In one of the most frequently used approaches for noncovalent immobilization of chemically unconjugated sugars, carbohydrate probes with various sizes and structures are spotted to nitrocellulose-coated glass slides using a microprinting device to generate microarrays. For chemically conjugated mono- and oligosaccharide immobilization, noncovalent fluorous-based microarray preparation is a useful technique. A fluorous tail is designed to aid saccharide purification and allow the direct formation of carbohydrate microarrays on appropriately prepared glass surfaces (Teflon/epoxy mixture). The resulting chips are beneficial for biological screening using various sugar-binding glycoproteins [79].

Covalent immobilization necessitates several chemical reaction steps and the selection of a proper linker. In comparison with the noncovalent immobilization techniques, covalent binding is usually more complex, sometimes requiring intensive synthesis work, but covalent binding of sugars offers high stability. As a wide range of applications requires high-stability arrays, in all these cases, the covalent binding should be considered. The physicochemical aspects of the Diels–Alder reaction for immobilization of biologically essential molecules have been extensively studied [80,81,82]. Self-assembled monolayers, where a quinone group reacts with cyclopentadiene for the immobilization of biomolecules, is an attractive and flexible method that is well-suited for tailoring peptide, carbohydrate, and small-molecule ligand monolayers. Notably, chemical and electrochemical modulation of quinone reactivity via its reduction to hydroquinone does not participate in the Diels–Alder reaction. This study provided new options to immobilize various biomolecules to gold surfaces. Based on this technology, mono- and oligosaccharide arrays were prepared by Diels—Alder-mediated immobilization of sugar–cyclopentadiene conjugates to form self-assembled monolayers of benzoquinone and Penta (ethylene glycol) groups. A simple method for preparing chemically unmodified carbohydrate microarrays on derivatized glass slides was described, along with its application in high-throughput screening of protein binding and pathogen detection [83]. This method is based on the use of the well-known reaction of aminooxy or hydrazide groups with free sugars as they complete site-specific and covalent immobilization of free carbohydrates on glass slides. This approach resulted in good stability, with covalent linkages between sugars and aminooxy or hydrazide groups on the surface.

Electrochemical glycan biosensors have been used for the analysis of glycan-binding proteins and viruses [84]. A glycan-immobilized field effect transistor biosensor was applied for the detection of influenza haemagglutinins. The field-effect transistor, which is a three-terminal semiconductor device, is based on a controlled input voltage. One study reported that a biosensor with an underivatized glycans immobilized biosensor on the aminooxy-modified silicon surface of the field-effect transistor device could detect 60 highly pathogenic asian avian influenza A (H5N1) virus (H5N1) or 6000 swine flu (H1N1) proteins, corresponding to 1 H5N1 virus or 12 H1N1 viruses with a dynamic range spanning nine orders of magnitude (ranging from attomolar to nanomolar concentration) [85]. The glycan-immobilized field-effect transistor biosensor is a promising device for the detection of several pathogenic viruses and bacteria via the glycan–protein interactions found ubiquitously in several infectious diseases. Glycan-immobilized field-effect transistor biosensors prepared on a silicon nanowire, with underivatized glycans covalently coupled via an aminooxy-modified surface, also successfully detected lectins down to 1 fM with a dynamic range of five orders of magnitude (ranging from femtomolar to nanomolar level). In addition, real-time monitoring of lectin binding has been reported [86].

Electrochemical impedance spectroscopy biosensor devices, based on electrodes modified by gold nanoparticles with thiolated glycans linked to nanoparticles through the sulfur–gold interaction backfilled with several thiols to stabilize a self-assembled monolayer of thiols on gold, successfully detected lectins down to 7 nM with a linear response within two orders of magnitude concentration [87]. A mannosylated polyaniline film was used for the impedimetric detection of a lectin in the range of 0.12–15 nM [88]. A recent study developed a glycan biosensor based on electrochemical impedance spectroscopy that can detect influenza haemagglutinins down to a single-molecule (attomolar) level, with a controlled density of sialylated glycans on the surface via the covalent immobilization of amine-modified glycan on a mixed self-assembled monolayer of –COOH groups. The working concentration range of the biosensor was attomolar to nanomolar for both lectins and influenza haemagglutinins [89].

Glycan biosensors made by anthraquinone-modified glycans linked to a graphene surface through π–π stacking interactions were used in the analysis of intact bacterial and cancerous cells. In this case, cyclic voltammetry was applied to detect changes in the redox potential of the quinone part as a redox probe upon interaction with an analyte. The HepG2 cell line was detected down to a concentration of 5000 cells/mL with a narrow dynamic range. Researchers were successful in the production of biomimetic interfaces densely clustered with sugar ligands able to sensitively capture glycoprotein receptors on live cancer cells through electrochemical approaches and with economic advantage [90]. A microcantilever glycan biosensor was used for the detection of an anti-viral protein, cyanovirin-N, down to 91 pM via a gold electrode modified by glycans through thiol–gold surface chemistry, with a dynamic range covering two orders of magnitude [91]. The three different types of glycan used in this study were trimannoside, nonamannoside, and galactoside. The best performance obtained was with the device patterned by nonamannoside [91]. Three *Escherichia coli* strains in solution were detected with as few as eight cells on a cantilever surface, with a wide dynamic range covering five orders of magnitude [92].

### 4.2. Lectin-Based Biosensors

Their specific binding affinity to carbohydrates has led to the usage of lectin proteins for the development of electrochemical and optical sensors. Examples include glucose- and mannose-specific lectin protein concanavalin A (Con A) lectin, which has been widely used in biosensor development. Con A plays an important role in the development of glucose, cancer cell, pathogenic bacteria, and hydrogen peroxide sensors due to its capability to immobilize glucose oxidase (GOx) and horseradish peroxidase (HRP) enzymes. Since these enzymes intrinsically contain hydrocarbon chains, they can be immobilized without labeling. Several studies have reported that bienzyme sensors consisting of GOx and HRP were successfully prepared, based on the layer-by-layer deposition of Con A and enzymes on the surface of the electrode and the electrochemical response to aromatic amines, phenols, and sulfides [93,94]. Electrodes can be modified with Con A, where specific substrates are adsorbed via strong affinity toward the hydrocarbon chains of these substrates. Nanomaterials are also used in biosensors to enhance the lectin-specific signal via amplification. Glycoprotein expression on living cells is another an emerging application of lectin-based biosensors [95].

Optics-based methods are well-suited for kinetic/affinity analysis of the interactions and detection of biomolecules in many areas, including immunochemistry, biomedicine, and diagnostics. Among these, surface plasmon resonance (SPR), intensively developed over the last two decades, is one of the most appropriate methods for fast and reliable label-free real-time biosensing with an immobilized ligand. Optical biosensors have been used for the analysis and glycoprofiling of a broad spectrum of analytes, such as lectins, glycoproteins, biomarkers, stem cells, and viruses. Real-time and label-free interactions are other examples of optical biosensors’ uses in biomedical diagnostics, such as antigen–antibody or lectin–saccharide affinity analysis. This function of SPR allows the technique to be used for automated monitoring of affinity interactions [96].

In recent years, biosensors have played an increasingly important role in the detection of bacteria due to their portability, sensitivity, and selectivity. Recently, the SPR technique has attracted extensive attention in the design of biosensors due to being label-free and less time-consuming than other methods [97]. Compared with quartz-crystal microbalance (QCM) or electrochemical measurements, SPR has some prominent advantages: firstly, it can provide real-time monitoring of biomolecular interactions; secondly, it can determine both kinetics parameters assaociation rate constant (ka) and dissociation rate constant (kd) and the thermodynamic parameters, including constant affinity (KA), which is useful for analyzing the biological specificity and functions of bacteria and lectins. A novel SPR biosensor was developed by Wang et al. [98] using lectin as a bioreceptor, employing multiple lectins for the detection of selective interactions with *E. coli* O157:H7. SAM of lectins was prepared to capture bacterial cells. The authors successfully achieved a 3 × 10^3^ CFU mL^-1^ limit of detection for the lectin from *Thymus vulgaris*. The sensitivity and reliability of this type of biosensor were found to be suitable for food safety analysis and the detection of *E. coli* O157:H7 in real food samples [98].

Another example of label-free bacterial detection includes electrochemical impedance spectroscopy (EIS), such as carbohydrates from microbe surfaces being targeted via selective attachment of lectins. Gamella et al. [99] reported biotinylated Con A and *E. coli* complex formation and immobilization of the lectin–bacteria complex on a gold screen-printed electrode (SPE), which only occurred in the presence of Con A. The detection was achieved via the changes in electron transfer of the complexes formed between multiple lectins and different bacterial species, including *Staphylococcus aureus, E. coli*, and *Mycobacterium phlei*. The use of small amounts of test solution and cheap chemicals helps in minimizing assay costs. Con A showed similar affinity toward *S. aureus* and *E. coli*, which posed a challenge for differentiation that was successfully resolved by measuring β-galactosidase activity for the surface-attached bacteria [99].

A magnetic-bead-based assay was used for bacterial cell capture and concentration determination via scanning electron microscope and fluorescence imaging [100]. Lin et al. [83] developed a wireless magnetoelastic sensor for *E. coli* O157:H7 in solution using Fe_3_O_4_ modified with mannose and Con A. The concentration of *E. coli* was observed via a change in the resonance frequency of the sensor upon bacterial binding. In another study, the electrochemical signals of bacteria sensors were employed for detection of *Deslforibrio caledoiensis* (in a bacterial solution) using modified nanoparticles (Fe_3_O_4_/MnO_2_) with Con A (biorecognition elements) and ferrocene (signaling molecule). Marine bud of Bohai Sea, China was the source used for the seed of D. caledoiensis [101].

### 4.3. Challenges for Glycan- and Lectin-Based Biosensors

The limited ability to work in a multiplexed format is the main problem with glycan/lectin biosensors. Ongoing research is required to examine electrochemical approaches that work in an array format, which may be possible since there are commercial electrodes found within ELISA plates (i.e., 96 electrodes) [102]. Glycan/lectin biosensors are expected to be more applicable for diagnosis than for initial analysis of a large sample set, for which the microarray format of determination is typical. A further challenge ahead for glycan/lectin biosensor application is the analysis of real complex samples; however, this task is already being tackled. Although controlled orientation during glycan immobilization can be easily achieved using glycan derivatives (i.e., one terminal–NH_2_ group for covalent amine coupling), controlled lectin immobilization intended for creating lectin biosensors and lectin microarrays is challenging due to the greater complexity of a protein molecule compared with that of a glycan molecule. Recent developments in reagent-coating of microchips [103,104] could provide possibilities for glycan/lectin coating inside miniaturized devices via optical detection.

## 5. Conclusions and Future Perspectives

Efficient and timely identification of causative agents is the most effective method to overcome the deadly consequences caused by infectious diseases, such as millions of deaths worldwide every year (approximately 20 million). Identification of pathogens may take a few days using conventional approaches; therefore, interest is growing in alternate methods, such as glycoprotein-based diagnostics, which can be exploited to develop rapid and economical methodologies by detecting surface located glycoproteins or their patterns with high specificity and selectivity. In this review, the biochemistry of glycoprotein, molecular interactions between glycoproteins and glycoprotein-binding molecules for the efficient and rapid detection of various pathogens the manufacture of biosensors based on selective complexation between glycoprotein lectins and carbohydrate chains were discussed as a future approach.

The crucial challenge is developing cost-effective and simple-to-use technologies for healthcare and diagnostics purposes. To this end, lectin-based biosensors offer selectivity between lectin protein and carbohydrates, immobilization of proteins labelled with sugars on optical transducers, and electrochemical sensors in mild conditions. The advantages of the lectin–carbohydrate affinity in manufacturing biosensors are that immobilization of any sugar-tagged proteins is possible on the surface of electrochemical and optical transducers or microchips, and the ability of lectins to bind to multiple glycan receptors on host cells can be detected. In other words, lectins are used not only as molecular glue for protein immobilization but also serve as a recognition element of biosensors. Furthermore, nanomaterials are becoming useful tools for amplifying the output signals of biosensors via accumulation of optically and electrochemically active compounds on the sensors. Detection of pathogenic bacteria using lectin-modified electrodes is based on the ability of lectin proteins to selectively bind to carbohydrate chains located on the surfaces of bacteria. It is estimated that approximately 54,000 individuals die globally each day due to infectious diseases; hence, assessing risk in real-time and detecting such pathogens, especially emerging pathogens like SARS-CoV-2, from asymptomatic individuals or infected/contaminated sites will help to improve the response to such global pandemics.

## Abbreviations

SARS-CoV-2	Severe acute respiratory syndrome coronavirus 2
ELISA	Enzyme-linked immunosorbent assay
PCR	Polymerase chain reaction
SAMs	Self-assembled monolayers
O-GlcNAc	O-linked glycoprotein β-N-acetylglucosamine
OST	Oligosaccharyltransferase
CPS	Capsular Polysaccharides
LPS	Lipopolysaccharides
ECA	Enterobacterial common antigen
HIV	Human immunodeficiency virus
HCV	Hepatitis C virus
MVN	Microvirin
VEE	Venezuelan equine encephalitis
SPR	Surface plasmon resonance
Leb	Lewis-b
GOx	Glucose Oxidase
QCM	Quartz crystal microbalance
SPE	Screen-printed electrode
